# Relationship between Cardiovascular Disease Risk and Neck Circumference Shown in the Systematic Coronary Risk Estimation (SCORE) Risk Model

**DOI:** 10.3390/ijerph182010763

**Published:** 2021-10-14

**Authors:** Serkan Asil, Ender Murat, Hatice Taşkan, Veysel Özgür Barış, Suat Görmel, Salim Yaşar, Murat Çelik, Uygar Çağdaş Yüksel, Hasan Kutsi Kabul, Cem Barçın

**Affiliations:** 1 Department of Cardiology, Gülhane Training and Research Hospital, 06010 Ankara, Turkey; dr_ender@outlook.com (E.M.); haticetaskan@gmail.com (H.T.); suatgormel@yahoo.com (S.G.); dr.salimyasar@hotmail.com (S.Y.); drcelik00@hotmail.com (M.Ç.); ucyuksel@hotmail.com (U.Ç.Y.); hkkabul@yahoo.com (H.K.K.); cembarcin@yahoo.com (C.B.); 2 Department of Cardiology, Gaziantep Dr. Ersin Arslan Training and Research Hospital, 27010 Gaziantep, Turkey; veyselozgurbaris@gmail.com

**Keywords:** neck circumference, anthropometry, waist circumference, cardiometabolic risk factors, cardiovascular risk score

## Abstract

Introduction: The most important way to reduce CVD-related mortality is to apply appropriate treatment according to the risk status of the patients. For this purpose, the SCORE risk model is used in Europe. In addition to these risk models, some anthropometric measurements are known to be associated with CVD risk and risk factors. Objectives: This study aimed to investigate the association of these anthropometric measurements, especially neck circumference (NC), with the SCORE risk chart. Methods: This was planned as a cross-sectional study. The study population were classified according to their SCORE risk values. The relationship of NC and other anthropometric measurements with the total cardiovascular risk indicated by the SCORE risk was investigated. Results: A total of 232 patients were included in the study. The patients participating in the study were analysed in four groups according to the SCORE ten-year total cardiovascular mortality risk. As a result, the NC was statistically significantly lower among the SCORE low and moderate risk group than all other SCORE risk groups (low-high and very high 36(3)–38(4) (IQR) *p*: 0.026, 36(3)–39(4) (IQR) *p* < 0.001, 36(3)–40(4) (IQR) *p* < 0.001), (moderate-high and very high 38(4) vs. 39(4) (IQR) *p*: 0.02, 38(4) vs. 40(4) (IQR) *p* < 0.001, 39(4) vs. 40(4) (IQR) *p* > 0.05). NC was found to have the strongest correlation with SCORE than the other anthropometric measurements. Conclusion: Neck circumference correlates strongly with the SCORE risk model which shows the ten-year cardiovascular mortality risk and can be used in clinical practice to predict CVD risk.

## 1. Introduction

Cardiovascular diseases (CVD) are the most common cause of mortality and morbidity worldwide [1]. It is generally believed that even though genetic defects underlie some infrequent forms of heart disease, most CVD is due to interactions between several gene variants and lifestyle factors. Although the specific contribution of the genes and the environment remains poorly understood, it is thought that environmental factors and lifestyle play a more dominant role in CVD development. This belief is based on the results of many studies showings that, to a large extent, CVD could be prevented by maintaining a healthy lifestyle. For instance, data from the Nurses’ Health Study3 suggest that 82% of coronary events could be prevented by maintaining a healthy lifestyle [2]. Similarly, it was found that 62% of all coronary events might have been avoided if men had adhered to a low-risk lifestyle [3]. An understanding of how different domains of the environment, individually and collectively affect CVD risk could lead to a better appraisal of CVD, and aid in the development of new preventive and therapeutic strategies to limit the increasingly high global burden of CVD.

Although the prognosis of CVD improves with developing technology and drug treatments, CVD prevention and patient risk assessment are the most effective treatment and protection methods for public health [1]. There are well-defined risk factors for CVD. These include smoking, family history, obesity, hypertension (HT), diabetes mellitus (DM), hyperlipidaemia (HL), lack of exercise, and stress [4]. Furthermore, to these well-known risk factors, many additional risk factors such as environmental factors, air pollution, heavy metals, seasons effect, ultraviolet, harmful nutrients, socioeconomic environment have been defined [5,6]. However, none of these risk factors show total CVD risk, so some risk models have been developed to define the total CVD risk level and their routine use is recommended [1,7]. The best known among these are the Framingham risk score and the SCORE (Systematic Coronary Risk Estimation) risk models recommended by the European Society of Cardiology [1,7].

In addition to these risk models, some anthropometric measurements are known to be associated with total CVD risk, including body mass index (BMI) and waist circumference (WC) [8,9]. WC is a useful tool for measuring central obesity and metabolic syndrome and is the most important anthropometric measurement proven to show cardiovascular risk worldwide [10]. However, cultural and environmental factors may influence WC measurements [11]. The room temperature, clothes, and undressing can all hinder accurate measurement. Furthermore, dyspepsia might lead to inaccurately high measurements [11].

Aside from the limitations of WC measurement, we know that specific fat distribution patterns, especially upper body adiposity, influence CVD risk [12]. Upper body obesity creates a higher susceptibility to glucose resistance, HL, DM, and hypertriglyceridemia than lower body obesity [12]. The distribution of upper-body subcutaneous adipose tissue illustrated by NC has been studied as an indicator for cardiovascular risk and insulin resistance and has been shown to be associated closely with the biochemical components of metabolic syndrome [13]. This anthropometric index can also provide a non-invasive, simple, patient-friendly method for the prediction of CVD risk. Besides, it cannot be affected by clothes or traditional behaviours.

The use of novel anthropometric markers such as NC to assess cardiometabolic risk factors and to correlate them to CVD is of great interest. In recent years, many epidemiological studies have suggested that NC is closely related to WC and BMI, thus it is an important indicator for predicting risk factors for cardiovascular disease. Furthermore, neck circumference is closely related to glucose and lipid metabolism disorders, insulin resistance, etc, and is related to various components of metabolic syndrome [14,15]. So, some research suggests that, in addition to BMI and WC, neck circumference can be an independent predictor of metabolic risk [13,16]. However, there are few population reports on NC as a direct marker of cardiovascular disease risk and coronary events [15,16,17]. It is also unclear whether NC may be associated with the SCORE risk model, which is the main cardiovascular risk indicator. Based on these findings, this study aimed to investigate the association of NC with the SCORE risk model.

## 2. Method and Statistical Analysis

### 2.1. Method

**Figure 1:**
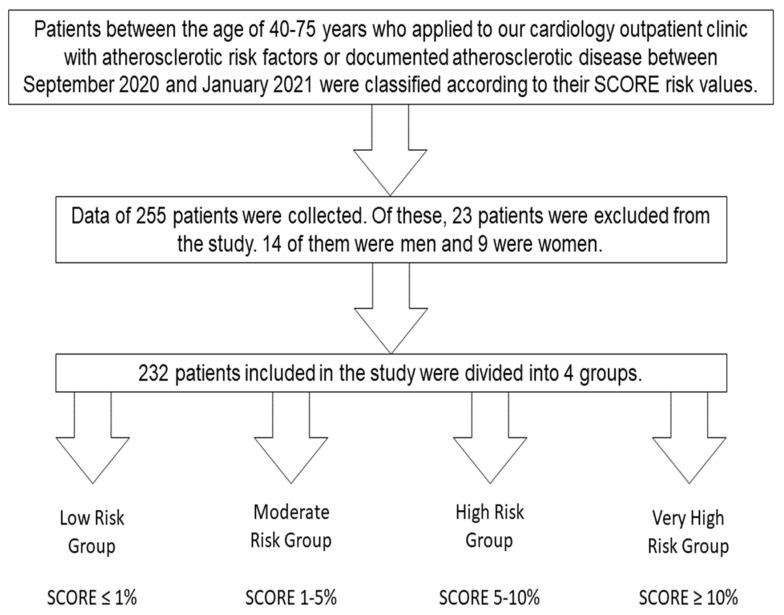
Flow diagram of the study design.

This was planned as a cross-sectional study. Patients between the ages of 40–75 years who applied to cardiology outpatient clinic with atherosclerotic risk factors or documented atherosclerotic diseases between September 2020 and January 2021 were classified according to their SCORE risk values (Figure 1). The relationship between patients’ neck circumference and other anthropometric measurements and their total cardiovascular risk indicated by the SCORE risk was investigated.

A total of 232 patients who were admitted to outpatient clinic during the study period were included. Detailed clinical history, physical examination, and laboratory examinations were performed for all patients and standard office blood pressure (BP) readings were recorded after 10 min of rest. While determining SCORE risk levels in patients with or without hypertension, office blood pressure measurements and, if necessary, ambulatory, and home blood pressure monitoring values were evaluated and mean blood pressure values were included. The SCORE risk levels of patients receiving antihypertensive therapy are recommended to be measured according to values under treatment, such as those who are not receiving therapy [1,18]. Similarly, while determining SCORE risk levels in patients with or without hyperlipidaemia, the current total cholesterol values were used irrespective of treatment [1]. The patients who did not receive treatment were evaluated according to the diagnostic and therapeutic algorithm recommended in the Dyslipidaemias guideline of the European Society of Cardiology [1,19].

Inclusion criteria included those aged 40–75 years, having cardiovascular risk factors such as HT, smoking, or HL, documented atherosclerotic cardiovascular disease, either clinical (DM, chronic kidney disease) or unequivocal on imaging (significant plaque on coronary angiography, computed tomography scan, or carotid ultrasound) [1]. Exclusion criteria included those with an active malignancy, decompensated heart failure, diseases that affect the neck circumference (thyroid disease, etc.), being bedridden, amputee, or having a condition that makes obtaining certain anthropometric measurements difficult.

Body weight, height, NC, and WC were the anthropometric measurements that were collected. Body weights were measured without shoes and with thin clothes using a calibrated electronic scale sensitive to 0.1 kg [20]. Height was measured by standing with feet side by side, heads, hips, and heels touching the wall, using the Frankfort method [20]. BMI (kg/m^2^) was calculated with the measured body weight and height. BMI values were classified according to the recommendations of the World Health Organization (WHO): below 18.5 kg/m^2^ as underweight, 18.5–24.99 kg/m^2^ as normal, 25.0–29.99 kg/m^2^ as overweight, 30.0–34.9 kg/m^2^ as obese, and ≥35 kg/m^2^ as morbidly obese [21]. WC was assessed by a single examiner, while the individuals were standing. Measurements were made at the midpoint between the iliac crest and the last rib using a 1.5 mm inelastic metric tape. WC was considered as at risk if ≥94 cm and at high risk if ≥102 cm for men and at risk if ≥80 cm and at high risk if ≥88 cm for women [21]. NC was measured by a single examiner, while the patient was standing in the Frankfurt position using an inelastic tape measure just below the head laryngeal protrusion [17]. Since no definite cut-off values have been established, men with NC < 37 cm and women with NC < 34 cm are likely to have a low BMI [13]. NC cut-off values of ≥39 cm for men and ≥35 cm for women are considered a metabolic syndrome predictor in the Turkish population [22].

In clinical practice, all existing recommendations on the prevention of atherosclerotic CVD propose assessing complete CVD risk. For this purpose, the European Society of Cardiology recommends the use of the SCORE risk model [1,19]. Countries are classified as high or low risk according to certain factors [1]. Turkey is considered among high-risk countries [1]. The following risk factors are used to calculate the risk: sex, age, smoking, systolic blood pressure, and total cholesterol [1]. While calculating the SCORE risk, the SCORE risk scale, and the internet application at www.heartScore.org have been used for high-risk countries.

The Declaration of Helsinki, which was updated in 2013, was followed in the protocol and the study was approved by the local ethics committee (University of Health Sciences, Gülhane Training and Research Hospital Ethics Committee, decision number and date: 2020-321, 4 September 2020).

### 2.2. Statistical Analysis

The SPSS software package was used for statistical analysis (Statistical Package for Social Sciences, version 22.0, SPSS Inc., Chicago, IL, USA). For quantitative data, mean, standard deviation, or median with interquartile range are given. The Kolmogorov–Smirnov test was used to verify the normal distribution and equality of variance expectations for all variables. For parametric data, one-way analysis of variance (ANOVA) was used, preceded by the Tukey test for post-hoc analysis, and for non-parametric results, Kruskal–Wallis analysis was used. The Chi-squared test was used to analyse categorical variables, and these are expressed as frequency and percentage. Statistical significance was described as a *p*-value of less than 0.05.

The primary aim of the study was to aim to investigate the association of NC with the SCORE risk model. In a previous study, 100 subjects were included and there was a strong correlation between NC and Framingham risk scores [23]. Based on these findings, alpha at 0.05, and power of 80%, we calculated that at least 88 patients had to be included in the study. Additionally, this number of patients was sufficient for subgroup (SCORE subgroups) analyses.

## 3. Results

A total of 232 patients were included in the study. The patients were analysed in 4 groups according to the SCORE ten-year total cardiovascular mortality risk. While 22.8% of the patients had a low SCORE risk, 27.2% had moderate, 19.8% high, and 30.2% very high risk. The analysis of the patients according to their basal demographic and clinical characteristics is given in Table 1. Specifically, we revealed that female patients were statistically significantly higher in the group with low SCORE ten-year total cardiovascular mortality risk compared to the other groups.

Comparison of patients according to laboratory parameters is given in detail in Table 2. Total cholesterol and LDL cholesterol values were found to be statistically significantly lower in patients with very high SCORE risk. This was attributed to the fact that 67.1% of very high-risk patients received statin therapy. HDL cholesterol levels were found to be statistically significantly lower in the group with very high SCORE risk compared to the low and moderate-risk groups (41(13) vs. 48 (8)-48 (13), *p* < 0.001). Triglyceride levels were statistically significantly higher in the group with high SCORE risk in most of the diabetic patient population compared to the other groups. This was thought to be due to the effect of DM on triglyceride levels.

The comparison of the groups according to their medical treatments is shown in Table 3. As expected, there were differences in medical treatments related to their risk levels and comorbidities.

When the groups were compared according to anthropometric and BP measurements, a statistically significant difference was found in body weight, BMI, WC, NC, and blood pressure (Table 4). Especially, body weight and BMI were found to be statistically significantly higher in groups with high and very high SCORE risk compared to the group with low SCORE risk. The median value (IQR) of neck circumference measurements according to the SCORE risk groups was 36(3), 38(4), 39(4), 40(4), respectively. According to these results, the relationship between SCORE risk values and neck circumference was found to be statistically significant between the low-risk group and all other groups (36(3) vs. 38(4) *p*: 0.026, 36(3) vs. 39(4) *p* < 0.001, 36(3) vs. 40(4) *p* < 0.001). Similarly, the relationship between SCORE risk values and neck circumference were found to be statistically significant between the moderate-risk group and the high and very high-risk groups (38(4) vs. 39(4) *p*: 0.02, 38(4) vs. 40(4), *p* < 0.001). Although there was a significant difference in blood pressure proportional to risk, the blood pressure values in the group with very high SCORE risk were found to be lower than the moderate and high SCORE risk groups. We thought that it was related to the high rate of use of drugs with antihypertensive activity in the very high SCORE risk group.

One of the most important results of this study is that it shows the correlation between the SCORE risk model and BMI, WC, and especially NC. A statistically significant weak correlation was found between BMI and WC and the SCORE risk model (*p* < 0.001 rho: 0.232, *p* < 0.001 rho: 0.210). A statistically significant moderate correlation was found between NC and the SCORE risk model (*p* < 0.001, rho: 0.527).

Statistically, significant variables associated with SCORE were evaluated in multiple logistic regression analyses. To perform multiple regression analysis in the SCORE risk model, the groups were combined into two groups. Low and moderate-risk patients were analysed in one group, and high- and very-high-risk patients in a different group. When the low-medium and high-very high groups were compared according to the SCORE risk model, only neck circumference was found statistically significant with age (Table 5).

## 4. Discussion

In this cross-sectional cohort study, we demonstrated the association between NC and the SCORE total 10-year CVD risk model, independent of and stronger than BMI and WC. The relationship between SCORE risk values and neck circumference were found to be statistically significant between the low-risk group and all other groups (36(3) vs. 38(4) *p*: 0.026, 36(3) vs. 39(4) *p* < 0.001, 36(3) vs. 40(4) *p* < 0.001). Similarly, the relationship between SCORE risk values and neck circumference were found to be statistically significant between the moderate-risk group and the high and very high-risk groups (38(4) vs. 39 (4) *p*: 0.02, 38(4) vs. 40(4), *p* < 0.001). Besides, in the multiple logistic regression analysis, we found that the stronger and statistically significant variable in the SCORE risk model among all variables was age and neck circumferences.

The use of novel anthropometric markers to assess cardiometabolic risk factors and link them to overall CVD risk is both significant and useful in clinical practice. Although WC and BMI have often been used to assess total CVD risk, they have many limitations and there are only a few population studies investigating NC as a predictor of cardiometabolic risk [13,16]. Some studies suggest that, in addition to BMI and WC, neck circumference can be an independent predictor of metabolic risk. [13,16]. However, no study has shown the relationship between the SCORE risk model and NC.

Body composition and CVD risk have been the subject of a lot of recent studies. For this purpose, WC and BMI are the most commonly used anthropometric measurements for the diagnosis of metabolic syndrome and the prediction of cardiovascular risk [10]. Typically, central obesity is linked to metabolic problems such as insulin resistance, diabetes, heart disease, and high triglycerides [24]. Although the association between visceral obesity and cardiometabolic abnormalities is well established, less is known about the metabolic relevance of other upper body subcutaneous fat stores. Upper body adipose tissue is an important contributor to circulating free fatty acids (FFA) and is more lipolytically active than lower body adipose tissue [25]. Because FFA concentrations are directly associated with insulin resistance, hepatic VLDL production, and endothelial dysfunction, upper body adipose tissue may have important cardiovascular and metabolic consequences [26,27,28].

As well, cultural and environmental influences can affect WC measurements. In this study, the relationship between WC, BMI, and the SCORE risk model was also investigated and a weak correlation was found. The fact that more parameters are affecting WC and BMI compared to NC may explain this weaker correlation between the SCORE risk model and NC. Additionally, upper body subcutaneous fat, as estimated by NC, may provide better risk prediction than WC and BMI [24].

Several studies have examined the correlation between NC and cardiometabolic risk factors in the past [12,13,16]. For example, studies show that NC is associated with insulin resistance, DM, HT, hypertriglyceridemia, high LDL cholesterol, and low HDL cholesterol [29,30]. The association between NC and insulin resistance and metabolic syndrome has also been investigated [29,31]. A study included in 2860 hypertensive patients have shown that in patients with hypertension, neck circumference is independently associated with cardiovascular risk factors [15]. Although the relationship between NC and independent cardiovascular disease risk factors has been demonstrated by these studies, there are only a few studies on risk models showing total cardiovascular mortality and morbidity risks. All these findings indicate that NC may be a simple method for determining total CVD risk.

Preis et al. assessed the association between increased NC and coronary artery disease risk factors in 3307 participants in a Framingham Heart Study cohort and concluded that NC was positively correlated with coronary artery disease risk [16]. Caro et al. examined 4607 patients according to Framingham Risk models and found that NC predicted the moderate and high-risk patient groups powerfully. This was one of the most important studies showing the relationship between a risk score showing total risk and NC [32]. In a different, smaller study, NC was shown to be closely associated with the 10-year risk of coronary artery disease as measured by the Framingham risk score [23].

SCORE is a risk estimate based on a large dataset of prospective European trials that estimates fatal atherosclerotic CVD events over ten years [1]. However, there is no study in the literature showing the relationship between SCORE risk and anthropometric measures or other parameters showing cardiovascular disease risk. In our study, it has been shown that the SCORE risk model correlates with BMI, WC, and NC, which has been proven to show the risk of cardiovascular and metabolic syndrome. The most significant of these results is the strong correlation between NC and the SCORE risk model, which is known to indicate a stronger relationship between upper body fat and cardiovascular risk. Although clinical and laboratory parameters such as diabetes mellitus, hypertension, cholesterol values, WC, BMI were significantly associated with the SCORE risk model in univariate analysis, only age and NC had a statistically significant relationship in multivariate logistic regression analysis. This result shows that NC can be a risk predictor as an easy-to-use anthropometric measurement that can be used in clinical practice. Studies have shown a relationship between the Framingham risk model and NC, this strong relationship with the SCORE risk model used in Europe is very important for clinical use.

Our study has many limitations that need to be considered. First, this was a small, single-centre study, lacking representation for the entire Turkish population. Furthermore, since our research focused solely on the Turkish community, our findings do not adequately reflect other cultures, environmental factors, or ethnic groups. Another important limitation of our study is that it is a cross-sectional study and we do not follow up the patient for the cardio-vascular endpoint. Therefore, further study is warranted considering these factors.

## 5. Conclusions

Our study showed us that neck circumference correlates strongly with the SCORE risk model and can be used in clinical practice to predict CVD risk. We hope that our research will pave a way for larger future studies, and we believe that anthropometric measurements such as neck circumference will be used more frequently in clinical use for CVD risk assessment.

## Figures and Tables

**Table 1:** Demographic and Clinical Characteristics of the Patients at Baseline.

Variables	Low Risk Group SCORE ≤ 1% *N* = 53	Moderate Risk Group SCORE 1–5% *N* = 63	High Risk Group SCORE 5–10% *N* = 46	Very High Risk Group SCORE ≥ 10% *N* = 70	*p*-Value
Female, %	74 ^a^	44	41	24	<0.001
Age, Median (IQR), years	47 (7) ^a^	55 (12) ^b^	55 (10)	59 (12)	<0.001
Married, %	74 ^c^	83	98	91	0.002
Smoking, %	25	38	28	27	0.38
Alcohol, %	27	40	20	13	0.46
Hypertension, %	36	40	59	41	0.108
Diabetes mellitus %	0 ^d^	0 ^d^	59 ^e^	29 ^e^	<0.001
Endocrine diseases, %	11	6	11	1	0.110
Previous TIA or stroke, %	0	2	0	0	0.441

IQR, interquartile range; TIA, transient ischemic attack. ^a^ There is a significant difference between low risk group and other groups. ^b^ There is a significant difference between moderate risk group and very high risk group. ^c^ There is a significant difference between low risk group and high- very high risk groups. ^d^ There is a significant difference between low -moderote risk group and high- very high risk groups. ^e^ There is a significant difference between high risk group and very high risk groups. Kruskal–Wallis analysis was used for Age, multiple chi-square test was used for other parameters.

**Table 2:** Comparisons of Biochemical Parameters Between SCORE Risk Groups.

Parameters	Reference Range	Low Risk Group SCORE ≤ 1% *N* = 53	Moderate Risk Group SCORE 1–5% *N* = 63	High Risk Group SCORE 5–10% *N* = 46	Very High Risk Group SCORE ≥ 10% *N* = 70	*p*-Value
Cholesterol, median (IQR), mg/dL	0–200	184 (50)	202 (62)	205 (71)	155 (77) ^a^	<0.001
LDL, median (IQR), mg/dL	60–160	110 (35)	119 (55)	124 (64)	80 (56) ^b^	<0.001
HDL, median (IQR), mg/dL	35–85	48 (13) ^c^	48 (8) ^d^	45 (11)	41 (13)	<0.001
Triglycerides, median (IQR), mg/dL	50–200	138 (79)	138 (84)	196 (177) ^e^	139 (120)	0.003
Uric acid, mean (SD), mg/dL	3.5–7.5	5.05 (1.06)	5.36 (1.31)	5.96 (1.20) ^f^	5.79 (1.55) ^g^	0.002
Serum albumin, median (IQR), g/dL	3.5–5.2	4.2 (0.3)	4.3 (0.4)	4.3 (0.4)	4.2 (0.4)	0.144
AST, median (IQR), U/L	15–40	18 (6) ^h^	21 (7)	21.5 (12.2)	21 (9.2)	0.006
ALT, median (IQR), U/L	10–40	16 (9.5) ^i^	20 (13)	23 (15.2)	20 (16.2)	0.007
eGFR_MDRD_, mean (SD)	>90	87.3 (16.8) ^j^	86.5 (15.2) ^k^	80.4 (13.5)	79.7 (11.6)	0.004

IQR, interquartile range; LDL, low-density lipoprotein; HDL, high-density lipoprotein; AST, aspartate transaminase; ALT, alanine transaminase; eGFR MDRD, estimated glomerular filtration rate modification of diet in renal disease; SD, standard deviation. ^a^ Comparisons of very high and moderate-high risk groups, *p*-value = 0.001. ^b^ Comparisons of very high and moderate-high risk groups, *p*-value = 0.001. ^c^ Comparisons of low and very high-risk groups, *p*-value = 0.001. ^d^ Comparisons of moderate and very high-risk groups, *p*-value = 0.002. ^e^ Comparisons of high and low- moderate risk groups, *p*-value = 0.003. ^f^ Comparisons of low and high-risk groups, *p*-value = 0.004. ^g^ Comparisons of low and very high-risk groups, *p*-value = 0.013. ^h^ Comparisons of low and high-risk groups *p*-value = 0.005. ^i^ Comparisons of low and high-risk groups *p*-value = 0.005. ^j^ Comparisons of low and very high groups *p*-value = 0.019.^k^ Comparisons of moderate and very high groups *p*-value = 0.031. ANOVA preceded by the Tukey test was used for eGFR and Uric Acid, Kruskal Wallis test was used for all other parameters.

**Table 3:** Comparisons of Medical Therapy Between SCORE Risk Groups.

Drugs	Low Risk Group SCORE ≤ 1% *N* = 53	Moderate Risk Group SCORE 1–5% *N* = 63	High Risk Group SCORE 5–10% *N* = 46	Very High Risk Group SCORE ≥ 10% *N* = 70	*p*-Value
ACE inhibitor or ARB, %	28.3 ^a^	31.7	56.5	64.3	<0.001
Beta-blocker, %	7.5	12.7	15.2	72.9 ^b^	<0.001
Calcium-channel blocker, %	17	14.3	28.3	11.4	0.109
Statin, %	1.9	1.6	15.2	67.1 ^b^	<0.001
OAD or insulin, %	0 ^c^	0 ^c^	56.5	27.1	<0.001

ACE, angiotensin converting enzyme; ARB, angiotensin II receptor blocker; OAD, oral antidiabetic. ^a^ There is a significant difference between low-risk group and high- very high-risk groups. ^b^ There is a significant difference between very high-risk group and other groups. ^c^ There is a significant difference between low and moderate risk group and other groups. Multiple chi-square test was used for all parameters.

**Table 4:** Anthropometric and blood pressure measurements of patients by SCORE risk groups.

Variables	Low Risk Group SCORE ≤ 1% *N* = 53	Moderate Risk Group SCORE 1–5% *N* = 63	High Risk Group SCORE 5–10% *N* = 46	Very High Risk Group SCORE ≥ 10% *N* = 70	*p*-Value
Weight, mean (SD), kg	79.72 (10.79)	82.43 (15.61)	86.57 (14.13) ^a^	84.84 (12.63) ^b^	0.006
Height, median (IQR), m	1.66 (0.14)	1.67 (0.15)	1.67 (0.09)	1.68 (0.13)	0.815
Body mass index, mean (SD), kg/m^2^	27.48 (4.38)	29.37 (5.43)	31.18 (5.63) ^c^	30.09 (3.80) ^d^	0.001
Neck circumference, median (IQR), cm	36 (3) ^e^	38 (4) ^f^	39 (4)	40 (4)	<0.001
Waist circumference, median (IQR), cm	97 (19) ^g^	102 (19)	103 (11)	105 (18)	0.009
Systolic blood pressure Median (IQR) mmHg	120.00 (19.50) ^h^	130.00 (22.00)	131.50 (31.00)	122.00 (30.00)	<0.001
Diastolic blood pressure Median (IQR) mmHg	78.00 (14.00) ^ı^	85.00(14.00)	85.00(12.75)	78.00 (10.25) ^j^	<0.001
Mean blood pressure Median (IQR) mmHg	91.33 (14.83) ^k^	100.00 (19.67) ^l^	101.83 (19.67)	92.00 (11.83)	<0.001

SD, standard deviation; IQR, interquartile range; BMI, body mass index. ^a^ Comparisons of low and high-risk groups, *p*-value = 0.007. ^b^ Comparisons of low and very high-risk groups, *p*-value = 0.02. ^c^ Comparisons of low and high-risk groups, *p*-value = 0.001. ^d^ Comparisons of low and very high-risk groups, *p*-value = 0.016. ^e^ Comparisons of low and moderate, high-very high-risk groups, *p*-value < 0.001. ^f^ Comparisons of moderate and high-very high-risk groups, *p*-value = 0.001. ^g^ Comparisons of low and high-very high-risk groups, *p*-value < 0.05. ^h^ Comparisons of low and moderate- high risk groups *p*-value < 0.001. ^ı^ Comparisons of low and high-risk groups *p*-values = 0.003. ^j^ Comparisons of very high risk and moderate-high risk groups *p*-values < 0.001. ^k^ Comparisons of low and high-risk groups *p*-values < 0.001. ^l^ Comparisons of low and moderate- high risk groups *p*-value: 0.017. ANOVA preceded by the Tukey test was used for weight and BMI, Kruskal–Wallis test was used for all other parameters.

**Table 5:** The result of multivariate logistic regression analysis for the prediction of the SCORE risk model.

Variables	Beta	Wald	*p* Value
Age	0.241	29.098	<0.001
Smoking	0.673	3.684	0.055
Hypertension	−0.564	0.977	0.323
Diabetes Mellitus	23.362	0.000	0.996
Cholesterol	−0.003	0.081	0.776
LDL Cholesterol	0.009	0.789	0.347
HDL Cholesterol	−0.36	1.096	0.295
Triglyceride	0.005	2.507	0.113
Body mass index	−0.092	2.923	0.87
Neck Circumference	0.544	20.804	<0.001
Waist Circumference	−0.056	3.173	0.75
Systolic blood pressure	−1.735	0.072	0.788
Diastolic blood pressure	−3.542	0.075	0.784
Mean blood pressure	5.273	0.074	0.785

LDL, low-density lipoprotein; HDL, high-density lipoprotein. To perform multiple regression analysis in the SCORE risk model, the groups were combined into two groups. Low and moderate-risk patients were analysed in one group, and high- and very-high-risk patients in a different group.

## Data Availability

The data are not publicly available due to ethical concerns, and we have not informed consent about the data availability statement.
